# Long non-coding RNA MIAT in development and disease: a new player in an old game

**DOI:** 10.1186/s12929-018-0427-3

**Published:** 2018-03-13

**Authors:** Cheng Sun, Lining Huang, Zhenglong Li, Kaiming Leng, Yi Xu, Xingming Jiang, Yunfu Cui

**Affiliations:** Department of Hepatopancreatobiliary Surgery, The Second Affiliated Hospital of Harbin Medical University, No.246 XueFu Avenue, Harbin, Heilongjiang Province China

**Keywords:** Long non-coding RNA, LncRNA, MIAT, Diseases

## Abstract

**Background:**

Long non-coding RNAs (lncRNAs), which are a portion of non-protein-coding RNAs (ncRNAs), have manifested a paramount role in the pathophysiology of human diseases, particularly in pathogenesis and progression of disease.

**Main body of the abstract:**

Myocardial infarction associated transcript (MIAT), which was recently found to demonstrate aberrant expression in various diseases, such as myocardial infarction, schizophrenia, ischemic stroke, diabetic complications, age-related cataract and cancers, is a novel disease-related lncRNA. This work summarize current evidence regarding the biological functions and underlying mechanisms of lncRNA MIAT during disease development.

**Short conclusion:**

LncRNA MIAT likely represents a feasible cancer biomarker or therapeutic target.

## Background

With advancements in global transcriptome profiling technique, the deregulation of lncRNAs has recently been demonstrated to be related to various human diseases, including most notably cancers [1, 2], neurological disorders [3], and cardiovascular diseases [4, 5]. Increasingly, some lncRNAs play a crucial role in cancer progression, such as proliferation, invasion and metastasis [6, 7]. Consequently, lncRNAs may be regarded as a promising marker for the prognosis of cancer [8]. According to the draft of the human genome project (HGP), the human genome contains only approximately 20,000 protein-coding genes, which accounts for less than 2% of the entire genome. Generally, at least 70% of the sequences are transcribed into RNAs in higher eukaryotic genomes. To our knowledge, most of these transcripts are ncRNAs, which were originally regarded as transcriptional noise and attracted limited attention [9].

LncRNAs are currently defined as a large and heterogeneous class of transcribed RNA molecules, which lack an open reading frame of significant length, and the length of lncRNA is greater than 200 nucleotides. Similar to protein-coding RNAs, the majority of lncRNAs are RNA polymerase II transcripts with a poly-A tail and 5′ cap. Interestingly, most lncRNAs are predominantly localized within the cell nucleus, and exhibit either lower evolutionary conservation or lower expression level than mRNAs [10–12]. Much of works have demonstrated that lncRNAs could regulate gene expression levels, post-transcriptional modifications, bind to transcription factors or miRNAs, and play modulator roles in many biological processes [13–15]. Thus, knowledge about their unique RNA properties, more tissue-specific expression fashion and more stable structure, it is expected that lncRNAs might advance our understanding of disease progression and unveil novel biomarkers for diagnosis and prognosis.

MIAT, also termed as Gomafu in human or Rncr2 in mouse [16–18], is a promising functional factor among all disease-associated lncRNAs, and exhibits deregulation in multiple diseases, including up-regulation in ischemic stroke, myocardial infarction, neuroendocrine prostate cancer, non-small-cell lung cancer, diabetic cardiomyopathy, cataract, chronic chagas disease cardiomyopathy, chronic lymphocytic leukemia and down-regulation in schizophrenia, diabetic nephropathy, bone disease. The present review summarizes current studies concerning the abnormal expression, biological function, molecular mechanism and clinical significance of MIAT in the initiation and progression of human diseases (Table 1).

**Table 1:** Functional characterization of MIAT in diseases

Disease type	Expression	Function	Related gene	Tissue	Animal	Role	Ref
Myocardial infarction	Up	Proliferation	miR-24/Furin/TGF-β1	Cardiac	Mouse	Pathogenesis	18, 31, 32
Schizophrenia	Down	Differention, maintenance	QKI/SRSF1/DISC1/ERBB4/DRD2/GRM3	Brain	Mouse	Pathogenesis	40–42
Ischemic stroke	Up	N.D.	N.D.	Blood	N.D.	Pathogenesis	37
Diabetic cardiomyopathy	Up	Apoptosis	miR-22-3p/DAPK2	Cardiac	Rat	Pathogenesis	56
Diabetic nephropathy	Down	Viability	Nrf2	Kidney	Rat	Pathogenesis	57
Cataract	Up	Proliferation, apoptosis Migration	miR-150-5p/Akt	Eye	N.D.	Pathogenesis	58
Bone disease	Down	Migration	N.D.	Adipose	N.D.	Pathogenesis	59
Chronic chagas disease cardiomyopathy	Up	N.D.	N.D.	Heart	Mouse	Pathogenesis	60
Chronic lymphocytic leukemia	Up	Proliferation Apoptosis	OCT4	Blood	N.D.	Pathogenesis	61
Neuroendocrine prostate cancer	Up	N.D.	N.D.	Prostate	N.D.	Oncogenic	47
Non-small-cell lung cancer	Up	Invasion	miR-150/ZEB1	Lung	N.D.	Oncogenic	52

*Abbreviation: N.D.* Not determined, *TGF-β1* transforming growth factor β1, *QKI* quaking homolog, *DISC1* disrupted in schizophrenia 1, *ERBB4* v-erb-a erythroblastic leukemia viral oncogene homolog 4, *DAPK2* Death Associated Protein Kinase 2

## Discovery and characterization of lncRNA MIAT

MIAT, which was initially reported in 2000 [19], is located at 22q12.1 with a length of 30,051 bp. Originally, through a large-scale case-control association study using 52,608 haplotype-based single nucleotide polymorphism (SNP) markers, Ishii et al. identified a susceptible locus for myocardial infarction. As a result, within this locus, they isolated a complete cDNA of a novel gene, designated MIAT [18]. In addition, this gene consists of five exons and all the splice junctions are considered to conform to the basic GT/AG rule [20, 21].

Though conventional sequence alignment searches did not identify any nonmammalian orthologous genes of MIAT [17], however, a search identified putative MIAT was orthologous to chick and Xenopus tropicalis. Interestingly, multiple clustered sequence repeats of the sequence ACUAACC were found in the largest exon of each orthologous gene, with anywhere from five to eight such repeats found within each transcript [22]. Furthermore, this repeat sequence includes the ACUAAY consensus recognition site for the RNA binding protein Quaking, which regulates both RNA subcellular localization and stability [23, 24], although the functional significance of this is unclear. In addition, unlike other nuclear-retained lncRNAs such as Xist and Air, MIAT shows no association with chromatin and is tightly associated with the nuclear matrix [17]. Generally, MIAT is highly conserved in placental mammals, and appears to be conserved back to amphibians [18, 25].

## LncRNA MIAT in human diseases

### Cardiovascular system diseases

#### Myocardial infarction

Myocardial infarction (MI), which accounts for the leading cause of death in the worldwide [26, 27], is characterized by myocardial remodeling processes involving left ventricular dilation, cardiomyocyte hypertrophy, arrhythmias, cardiac fibrosis, and cell death accompanied by altered expression of genes, leading to heart failure [28–30].

Constant researches have led to the fact that MIAT is involved in MI. Ishii et al. originally identified that the aberrant expression of one SNP rs2301523 was related to the pathogenesis of MI. Subsequently, through a large-scale case-control association study using 52,608 gene-based genome-wide tag SNPs, they found that this SNP in MIAT, due to its altered expression by the single nucleotide polymorphism, which had a significant association with the pathogenesis of MI [18]. Through the quantitative PCR technology, Vausort et al. found that Patients with STEMI (ST-elevation myocardial infarction) had lower levels of MIAT in peripheral blood cells compared to patients with NSTEMI. Of all the cardiovascular risk factors, the expression level of MIAT in MI patients was significantly associated with hypertension and smoking. Moreover, MIAT was positively associated with the percentage of lymphocytes and negatively associated with neutrophils and platelets in all the inflammation markers. In addition, according to their 4-month follow-up, the expression level of MIAT was a significant univariate predictor of LV dysfunction [31]. Qu et al. identified MIAT expression was remarkable up-regulated in a mouse model of MI heart compared with that in aninals contrals, Most notably, MIAT knokdown improved cardiac function and inhibited interstitial Fibrosis by inhibiting collagen production and cardiac fibroblasts proliferation [32]. These data suggest that MIAT may serve as a specific biomarker and therapeutic target for MI.

#### Ischemic stroke

Stroke is the leading cause of death and long-term disability in developed countries [33, 34]. Ischemic stroke (IS) is the most common form of stroke, accounting for approximately 87% of all cases [35, 36]. Thus, developing a noninvasive high-sensitive blood biomarker for early screening and monitoring of brain ischemia patients is feasible and valuable.

Zhu et al. indicated that MIAT expression level was significantly up-regulated in peripheral blood leukocytes of 189 IS patients compared with controls. Moreover, increased MIAT was markedly correlated with NIHSS scores, mRS, high-sensitivity C-reactive protein, and infarct volume in IS. In addition, the overall survival analysis showed that IS patients with higher MIAT expression had a relatively poor prognosis compared with the low group. Meanwhile, the multivariate analysis revealed that MIAT was an independent prognostic marker of functional outcome and death in patients with IS. Furthermore, ROC curves indicated that MIAT could serve as a potential marker for discriminating IS patients from the controls. [37].

### Nervous system diseases

#### Schizophrenia

Schizophrenia (SCZ) disorder, a debilitating mental disorder affecting about 1% of the population [38], is believed to be the result of combinations of genetic and environmental factors resulting in brain dysfunction [39]. Aprea et al. have demonstrated the physiological roles of MIAT in differentiation of neurons and excitatory neurons in embryonic brain for the first time [40]. Then, Barry et al. further revealed that MIAT was significant decreased at 1 and 3 h and a returned to normal levels by 5 h.in a neuronal stimulation time course using depolarized mouse primary cortical neurons. Moreover, MIAT was observed significantly decreased in depolarized hiPSC-derived neurons. In addition, cortical MIAT was found significantly reduced by 1.75-fold in SCZ of post-mortem superior temporal gyrus in relative to controls [41].Afterwards, Rao et al. observed that rs1894720, one of eight tag SNPs that covered the whole MIAT locus, which was significantly associated with paranoid schizophrenia in two independent Han Chinese schizophrenia case–control cohorts(discovery sample from Shanxi Province: 1093 patients with paranoid schizophrenia and 1180 control subjects; replication cohort from Jilin Province: 1255 cases and 1209 healthy controls) using a two-stage association analysis [42]. Hence, continuing and thorough exploration for MIAT may make a new breakthrough in the diagnosis and treatment of SCZ.

### Cancers

#### Neuroendocrine prostate cancer

Prostate cancer (PCa) represents the second most frequently diagnosed neoplasm and is the fifth leading cause of cancer death worldwide [43]. Between 0.5 and 2% of newly diagnosed prostate neoplasms are classified as neuroendocrine PCa (NEPC), which is insensitive to all forms of hormonal treatment [44], is often diagnosed at a metastatic stage [45]. In addition, no treatment has demonstrated efficacy in extending the survival of NEPC patients, while median NEPC survival is only 7 months [46]. Crea et al. discovered that MIAT was the most highly up-regulated transcript in 18 prostatic adenocarcinoma and three NEPC models. Moreover, unsupervised hierarchical clustering demonstrated that MIAT expression could efficiently discriminate NEPC and adenocarcinoma samples in a clinical cohort. In addition, oncomine database analyses revealed that MIAT expression was restricted to a small percentage of PCas, with high metastatic potential, poor prognosis and frequent Rb mutations [47]. Notably, all those are hallmarks of NEPC [48, 49]. In general, MIAT displays advantageous characteristics as a novel biomarker and as a therapeutic target in prostate cancer.

#### Non-small-cell lung cancer

Lung cancer remains the leading cause of death from cancer worldwide [50]. Non-small-cell lung cancer (NSCLC), which accounts for approximately 80% of all lung cancer cases, is the primary category of lung cancer, and its prognosis is poor despite recent progress in chemotherapy [51]. Therefore, an urgent need to develop reliable prognostic biomarkers and molecular targets aimed to reduce the morbidity and mortality associated with NSCLC. Zhang et al. reported that the expression of MIAT in 31 NSCLC tissues was up-regulated compared to mixed 30 normal tissues. Furthermore, MIAT expression was significantly higher in all human lung cancer lines compared to control group. In addition, knockdown of MIAT substantially inhibited the invasive ability of NSCLC cells [52]. Taken together, MIAT may therefore serve as a valuable prognostic biomarker and therapeutic target for NSCLC.

### Endocrine system **diseases**

#### Diabetes mellitus

Diabetes mellitus (DM) is commonly derived from the defects in insulin secretion or insulin action, or both of them and the chronic DM would induce the damage or dysfunction of several organs, such as heart, eyes, nerves, as well as kidney and blood vessels [53, 54]. Recently, MIAT emerged as a potential regulator for diabetic complications [55].

Diabetic cardiomyopathy (DCM), which is defined as myocardial dysfunction occurring in the absence of coronary artery disease and hypertension, carries a substantial risk for the subsequent development of heart failure. Zhou et al. demonstrated that the expression level of MIAT was up-regulated in the myocardium of diabetic rats and MIAT knockdown was found to inhibit apoptosis in cardiomyocytes exposed to high glucose. Moreover, decreased MIAT expression was shown to improve diabetic rats’ cardiac structure, such as LVEDD (left ventricular end-diastolic dimension) and LVESD (left ventricular end systolic diameter). In addition, LVFS (left ventricular fractional shortening), LVEF (left ventricular ejection fraction) and E/A ratio, indicators of left ventricular systolic and diastolic function, could be improved by down-regulated MIAT in diabetic rats [56].

Diabetic nephropathy (DN) is one of the most common diabetic complications that develops secondary to diabetes, represents the leading cause of chronic kidney failure, and it remains a major contributor to increase morbidity and mortality among individuals with diabetes [55]. Zhou et al. discovered that MIAT wan lowly expressed in renal tubule of diabetic rats compared with that in normal controls. Moreover, down-regulated MIAT was negatively related to serum creatinine and BUN in diabetic rats. In addition, expression level of MIAT was significantly decreased in human renal tubular epithelial cells due to the increase of glucose. Biologically, MIAT over-expression significantly improved human renal tubular cell viability in vitro experiment [57]. These data suggested that MIAT was important for high glucose induced organ injury. Hence, MIAT is expected to serve as a novel diagnostic biomarker and therapeutic target.

#### Other diseases

Shen et al. reported that MIAT level was significantly up-regulated in the plasma fraction of cataract patients, MIAT knockdown could affect the proliferation, apoptosis and migration of Human lens epithelial cells (HLECs) [58]. Jin et al. indicated the expression of MIAT was down-regulated in a time-dependent manner during human adipose-derived stem cells (hASCs) osteo-induction, MIAT knockdown promoted osteogenic differentiation of hASCs both in vitro and in vivo. Furthermore, decreased MIAT expression reversed the negative effects of TNFα on osteoblast differentiation in hASCs [59]. Frade et al. confirmed that MIAT was specially increased in heart tissue biopsies from chronic chagas disease cardiomyopathy (CCC) patients but not in those from dilated cardiomyopathy (DCM) or control tissue. Moreover, overexpression of MIAT was confirmed in mouse model of T.cruzi infection [60]. Sattari et al. reported that MIAT was increased in malignant mature B cells including established cell lines of chronic lymphocytic leukemia (CLL) and non-Hodgkin lymphoma origin as well as primary leukemic cells obtained from CLL patients. Furthermore, decreased MIAT expression inhibited cell proliferation compared to the growth of control treated cells. These date suggested that MIAT functioned to protect malignant mature B cells from cell apoptosis [61].

## Regulation mechanisms of MIAT

The competitive endogenous RNA (ceRNA) hypothesis has been proposed that protein-coding messenger RNAs and non-coding RNAs communicate with each other by competing for binding to shared miRNAs, a family of small non-coding RNAs responsible for the post-transcriptional regulation of gene expression [62]. The ceRNA regulatory network is a major mechanism of MIAT in disease development. Investigations had reported that MIAT, which worked as a ceRNA for miR-150-5p and constituted a MIAT/miR-150-5p/VEGF feedback loop, regulated endothelial and Müller cell function, such as proliferation, migration and apoptosis [55, 63]. Shen et al. found that MIAT acted as a ceRNA, and formed a MIAT/miR-150-5p/Akt feedback loop to regulate HLEC function, including proliferation, apoptosis and migration [58]. Qu et al. confirmed that MIAT functioned as a ceRNA for miR-24 to modulate Furin and TGF-β1 expression, suggested that a MIAT/miR-24/Furin/TGF-β1 modality participated in MI or other cardiac pathological processes associated with fibrosis [37]. Zhou et al. demonstrated that MIAT might function as a ceRNA to upregulate DAPK2 expression by sponging miR-22-3p, which consequently leaded to cardiomyocyte apoptosis involved in the pathogenesis of DCM [56]. Zhang et al. reported that MIAT could enhance the expression of ZEB1 by acting as a miR-150 sponge to promote cell invasion during NSCLC [52].

In addition to acting as a ceRNA, MIAT can also activate various signaling pathways. Zhou et al. discovered that MIAT over-expression enhanced Nrf2 (nuclear factor erythroid 2-related factor 2) expression in high glucose incubated renal tubular epithelial cell line HK-2, Furthermore, alteration of cellular MIAT expression level caused negatively expression of Nrf2 protein but had no effect on Nrf2 mRNA level. In addition, the MIAT was indicated to pro-stability act on Nrf2 expression in HK-2. Biologically, decreased MIAT expression reduced cell viability, whereas this inhibitory action was abrogated by Nrf2 overexpression. Hence, MIAT/Nrf2 signal axis might serve as an important signaling pathway for high glucose induced renal tubular epithelial injury [57]. Barry et al. revealed that MIAT strongly bound to the splicing regulator QKI and SRSF1 (serine/arginine-rich splicing factor 1) in nuclear compartment of SZ neuron. Furthermore, increased MIAT significantly decreased DISC1 (disrupted in schizophrenia 1), ERBB4 (v-erb-a erythroblastic leukemia viral oncogene homolog 4) and their alternatively spliced variants in SZ patient brains. In addition, the expression level of MIAT was related to dopamine (DRD2) and glutamate (GRM3) pathways, DRD2 splice-variant expression was reciprocally regulated by MIAT, GRM3 were upregulated by MIAT knockdown. Hence, MIAT expression might contribute to the pathophysiology of SZ by acting as a splicing factor scaffold for the SZ-associated splicing factor [36]. Sattari et al. showed that MIAT constituted positive feedback regulatory loop with its transcriptional regulator OCT4 to promote celluar proliferation in human malignant mature B cells [61]. These findings reveal the complexity of these interactions, which contribute to malignant transformation and disease progression.

## Conclusion and future perspectives

**Fig. 1:**
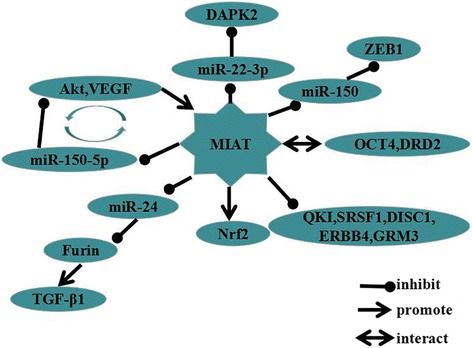
MIAT mediates mechanisms involved in disease progression

MIAT, a well-characterized disease-related lncRNA, can impact cellular functions such as proliferation, apoptosis, and invasion in various human diseases. The regulatory mechanisms of MIAT are extraordinarily complicated and involve multiple steps (Fig. 1). The aberrant expression of MIAT exhibits a critical role in disease development, and may serve as a potential biomarker for diagnosis and prognosis. However, the chemical stability and expression levels of MIAT in biological specimens have not been clearly validated. Due to its strong disease specificity and reduced systemic toxicity, MIAT, which acts as a viable therapeutic target, is extremely promising. Taken together, the research that focuses on MIAT is still in the early stage, and some pivotal matters for its clinical application still need to be resolved. The detailed regulatory mechanisms upstream and downstream of MIAT should be paid attention to exploring, and consolidate the underlying mechanisms from the former. Undoubtedly, efforts to clarify the underlying mechanisms promise that MIAT will ultimately reach the clinic.

## Abbreviations

BUN: Blood urea nitrogen
CCC: Chronic chagas disease cardiomyopathy
ceRNA: Competitive endogenous RNA
CLL: Chronic lymphocytic leukemia
DCM: Diabetic cardiomyopathy
DISC1: Disrupted in schizophrenia 1
DM: Diabetic mellitus
DN: Diabetic Nephropathy
ERBB4: v-erb-a erythroblastic leukemia viral oncogene homolog 4
hASCs: Human adipose-derived stem cells
HF: Heart failure
HGP: Human genome project
IS: Ischemic stroke
lncRNAs: Long non-coding RNAs
LV: Left ventricular
LVEDD: Left ventricular end-diastolic dimension
LVEF: Left ventricular ejection fraction
LVESD: Left ventricular end systolic diameter
LVFS: Left ventricular fractional shortening
MI: Myocardial infarction
MIAT: Myocardial infarction associated transcript
ncRNA: Noncoding RNA
NEPC: Neuroendocrine PCa
Nrf2: Nuclear factor erythroid 2-related factor 2
NSCLC: Non-small-cell lung cancer
PCa: Prostate cancer
SCZ: Schizophrenia
SNP: Single nucleotide polymorphism
SRSF1: Serine/arginine-rich splicing factor 1
STEMI: ST-elevation myocardial infarction
ZEB1: Zinc finger E-box binding homeobox 1
